# Point-of-care cell therapy manufacturing; it’s not for everyone

**DOI:** 10.1186/s12967-022-03238-5

**Published:** 2022-01-15

**Authors:** David F. Stroncek, Robert P. T. Somerville, Steven L. Highfill

**Affiliations:** Center for Cellular Engineering, Department of Transfusion Medicine, NIH Clinical Center, 10 Center Drive-MSC-1184, Building 10, Room 1C711, Bethesda, MD 20892-1184 USA

**Keywords:** Cell therapy, Gene therapy, Cancer immunotherapy, Point-of-care manufacturing, Decentralized manufacturing

## Abstract

The use of cellular therapies to treat cancer, inherited immune deficiencies, hemoglobinopathies and viral infections is growing rapidly. The increased interest in cellular therapies has led to the development of reagents and closed-system automated instruments for the production of these therapies. For cellular therapy clinical trials involving multiple sites some people are advocating a decentralized model of manufacturing where patients are treated with cells produced using automated instruments at each participating center using a single, centrally held Investigational New Drug Application (IND). Many academic centers are purchasing these automated instruments for point-of-care manufacturing and participation in decentralized multiple center clinical trials. However, multiple site manufacturing requires harmonization of product testing and manufacturing in order to interpret the clinical trial results. Decentralized manufacturing is quite challenging since all centers should use the same manufacturing protocol, the same or comparable in-process and lot release assays and the quality programs from each center must work closely together. Consequently, manufacturing cellular therapies using a decentralized model is in many ways more difficult than manufacturing cells in a single centralized facility. Before an academic center decides to establish a point-of-care cell processing laboratory, they should consider all costs associated with such a program. For many academic cell processing centers, point-of-care manufacturing may not be a good investment.

## Commentary

Cell and gene therapies have been produced and used for early phase clinical trials in academic health centers for more than 30 years [1]. The variety and quantity of cellular therapies produced was limited until the remarkable clinical success of the cancer immunotherapy Chimeric Antigen Receptor (CAR) T-cells. Since then the field has grown extraordinarily in terms of the type and quantity of cellular therapies used clinically [1]. In fact, a number of CAR T-cell therapies used for the treatment of B-cell malignancies have been licensed by regulatory agencies and are being produced commercially to target CD19 (Yescarta, Kymriah, Tecartus, Breyanzi) and, more recently, BCMA (Abecma).

This success has led to the development and increased availability of reagents for manufacturing clinical cellular therapies, and a number of suppliers now have entire sections strictly devoted to T-cell therapy. In addition, closed-system automated instruments have been developed for the production of cellular therapies [2]. These instruments are very attractive to academic institutions since their use may significantly reduce the need for cleanroom space, and hence lower the overall cost of manufacturing. The nature of these automated instruments and off-the-shelf Good Manufacturing Practices (GMP) reagents makes it feasible for academic laboratories originally designed and set up to process Hematopoietic Stem Cells (HSC) for transplantation to produce more advanced cellular therapies. In fact, many academic centers are purchasing these instruments and have begun to manufacture CAR T-cells and virus specific T-cells.

Along with chemotherapy and HSC transplantation, CAR T-cells have become an important tool for treating B-cell leukemia and lymphoma. The availability of GMP reagents and automated instruments have led some people to advocate for the point-of-care production of CAR T-cells by hospitals using these automated instruments. Although making CAR T-cells using these instruments is relatively easy, meeting the quality, safety, potency and regulatory expectations is not. While the processing and clinical use of HSCs for transplantation are subjected to few regulations, cell and gene therapies, including CAR T-cells, are recognized as ‘living-drugs’ regulated under the Food and Drug Administration’s Center for Biologics Evaluation and Research (CBER), and are expected to meet strict quality standards similar to those applied to standard drugs regulated under the Center for Drug Evaluation and Research (CDER). In line with the explosion in the number of CAR T-cell therapy Investigational New Drug Application (IND) submissions, so too has there been ever-increasing regulatory requirements that need to be satisfied prior to approval. Because of this, a robust quality management system is a requisite to ensure that all therapies produced are consistently of high quality and are safe.

For multicenter cellular therapy clinical trials some people are also advocating a “decentralized” model of manufacturing where patients are treated with cells produced using point-of-care manufacturing at each participating center on a single, centrally held IND. Multiple site manufacturing requires harmonization of product testing and manufacturing in order to interpret the results of the clinical trial. While this seems simple, it’s quite challenging since all centers should use the same manufacturing protocol, the same or comparable in-process and lot release assays, and the quality programs from each center must work closely together. In many respects this requires more effort than manufacturing cellular therapies at a single location for multiple centers.

Regulatory agencies are receptive to local or decentralized manufacturing [3] and this approach makes sense for some cellular therapies. For example, the cancer immunotherapy Tumor Infiltrating Lymphocytes (TIL) involves the infusion of up to 100 billion cells which are given to patients immediately after they are harvested. Since it is not feasible to transport large quantities of TIL which are not cryopreserved, manufacturing TIL in one centralized laboratory for administration at multiple clinical sites is not yet practical. While manufacturing TIL at multiple centers may be necessary, it will require significant coordination and strict oversight and likely considerable expense.

While the availability of highly automated instruments and GMP reagents for manufacturing cell therapies is an important advancement, the process of providing clinical cell and gene therapies remains complex. Providing these therapies is even more complex for centers participating in multiple center clinical trials which involve decentralized manufacturing. Furthermore, most academic centers are manufacturing cell therapies for early phase clinical trials which are of limited duration. If early trials find that a cell therapy is safe and suggests that it is clinically effective and if it is to be continued to be used clinically, the product must move to advanced clinical trials and, eventually, a Biologics License Application (BLA). Regulatory requirements are even more strict for producing cell therapies for late phase clinical trials and for producing licensed products. Before an academic center decides to establish a clinical cell processing laboratory, they should consider all costs associated with such a program along with current and future needs and applications. For many academic cell processing centers, point-of-care manufacturing may not be a good investment.

## Abbreviations

BLA: Biologics License Application
CBER: Center for Biologics Evaluation and Research
CDER: Center for Drug Evaluation and Research
CAR: Chimeric Antigen Receptor
GMP: Good Manufacturing Practices
IND: Investigational New Drug Application
TIL: Tumor Infiltrating Lymphocytes
HSC: Hematopoietic Stem Cells
